# Cardiovascular disease and depression as mediators between red blood cell distribution width to albumin ratio and cognitive impairment in older adults

**DOI:** 10.3389/fphys.2025.1587635

**Published:** 2025-06-11

**Authors:** Hui Wang, Xinyu Bai, Cong Wang, Sensen Wu, Dikang Pan, Lianrui Guo, Peng Yu, Jianming Guo, Yongquan Gu

**Affiliations:** ^1^ Department of Vascular Surgery, Xuanwu Hospital, Capital Medical University, Beijing, China; ^2^ Department of Ophthalmology, The Second Hospital of Jilin University, Changchun, Jilin, China

**Keywords:** red blood cell distribution width/albumin ratio, cognitive impairment, NHANES, older adults, cardiovascular disease, depression

## Abstract

**Background:**

Cognitive impairment is a major public health concern in aging populations, and early identification of risk factors is critical. The red blood cell distribution width to albumin ratio (RAR) has emerged as a potential biomarker reflecting inflammatory and nutritional status, but its association with cognitive impairment remains unclear.

**Objective:**

This study investigates the relationship between RAR and cognitive impairment in older adults, and explores potential mediating variables that may influence this association.

**Methods:**

A total of 2,913 participants aged ≥60 years from the National Health and Nutrition Examination Survey (NHANES) 2011–2014 cycles were analyzed, including 1,291 with cognitive impairment. Logistic regression assessed the association between RAR and cognitive impairment, adjusting for potential confounders such as age, gender, race, education, marital status, weight, height, and comorbidities. Restricted cubic spline (RCS) analysis evaluated the dose-response relationship and identified nonlinear thresholds. Subgroup analyses explored interactions between RAR and demographic/clinical factors. Causal mediation analysis, using a generalized linear model with a probit link and adjusting for age, sex, race, and education, was performed to estimate total, direct, and indirect effects via bootstrap resampling.

**Results:**

RAR was positively associated with cognitive impairment (P < 0.05). RCS analysis revealed a nonlinear threshold, with RAR ≥3.2 significantly increasing the risk of cognitive impairment (OR = 1.24, 95% CI: 1.11–1.38, P < 0.001). Subgroup analysis showed significant interactions between RAR and cardiovascular disease (CVD), hypertension, and depression (P for interaction <0.05). Stratified analysis found a stronger association between RAR and cognitive impairment in individuals without hypertension, CVD, or depression. Mediation analysis indicated that CVD (P = 0.036) and depression (P = 0.032) partially mediated the relationship, with CVD explaining 4.49% of the total effect. Hypertension had no significant mediating effect.

**Conclusion:**

RAR is significantly associated with cognitive impairment, with a stronger association when RAR ≥3.2. CVD and depression partially mediate this relationship, suggesting RAR as a potential biomarker for cognitive impairment in older adults.

## Introduction

Cognitive impairment is a prevalent and critical health concern affecting the global elderly population. As population aging accelerates, it has emerged as a pressing public health challenge. The World Health Organization estimates that nearly half of individuals aged 65 and older experience some level of cognitive decline, with approximately 10% at risk of advancing to cognitive impairment (Langa and Levine, 2014; Pérez et al., 2022). Cognitive impairment arises from a variety of causes and is frequently linked to factors such as aging, genetic predisposition, lifestyle choices, and chronic health conditions. Chronic diseases, including cardiovascular disease (CVD), diabetes, and hypertension, have been shown to have a strong association with an increased risk of cognitive decline (Biessels and Despa, 2018; Hainsworth et al., 2024; Santisteban et al., 2023; van der Flier et al., 2018). Additionally, lifestyle factors such as healthy dietary practices, regular physical activity, active social engagement, and good sleep quality have been demonstrated to play a beneficial role in preserving cognitive function (Biessels and Whitmer, 2020; Petersen et al., 2018).

Growing evidence suggests that systemic inflammation and malnutrition play central roles in cognitive decline, possibly through mechanisms such as oxidative stress, blood–brain barrier disruption, and endothelial dysfunction. Biomarkers that reflect both inflammation and nutritional status may offer insight into early cognitive deterioration. One such biomarker is the red blood cell distribution width to albumin ratio (RAR), which integrates hematologic and nutritional status and may be linked to cerebrovascular and neurodegenerative processes (Li et al., 2024; Li and Xu, 2023; Seo et al., 2022). Red blood cell distribution width (RDW), which measures the standard deviation of red blood cell volume distribution, indicates the variability in red blood cell size. Research has demonstrated that increased RDW levels are linked to several conditions, including anemia, inflammation, and CVD (Montagnana et al., 2011; Turcato et al., 2016; Wang and Xu, 2024; Winchester et al., 2018). Albumin (ALB), a protein produced by the liver, is essential for maintaining colloid osmotic pressure in the bloodstream. Reduced albumin levels are often indicative of malnutrition, liver dysfunction, or chronic diseases (Min et al., 2022; Murayama et al., 2017). The RAR integrates the attributes of RDW and albumin, providing a more holistic measure of a patient’s health status (Ma et al., 2024; Shan et al., 2024). Recent studies suggest that elevated RAR may contribute to cognitive decline through multiple biological pathways. Increased RDW reflects heightened systemic inflammation and oxidative stress, both of which can exacerbate neuroinflammatory responses and lead to neuronal damage (Salvagno et al., 2015; Hong et al., 2020). Concurrently, low serum albumin levels may result in endothelial dysfunction, compromise the integrity of the blood–brain barrier, and weaken the body’s antioxidant defenses (Alzayadneh et al., 2023; Belinskaia et al., 2021). These alterations collectively impair cerebral blood flow, increase vascular permeability, and promote the accumulation of neurotoxic substances—processes that may accelerate the progression of cognitive deterioration. Based on the above evidence, we hypothesize that elevated RAR may be associated with cognitive impairment through mechanisms such as enhanced inflammation, increased oxidative stress, and blood–brain barrier dysfunction, and aim to explore potential mediators that may influence this relationship.

The clinical applications of the RAR are becoming increasingly widespread. As a simple and cost-effective indicator, RAR can assist physicians in assessing disease risk, predicting patient prognosis, and monitoring treatment response. For instance, elevated RAR levels are often associated with poorer clinical outcomes in patients with chronic conditions such as cardiovascular diseases, diabetes, kidney disease, and cancer (Chen et al., 2024; Hao et al., 2024; Zhao et al., 2022). RAR effectively reflects factors that impact cerebrovascular health, enhance vascular permeability, increase neuroinflammatory responses, and reduce antioxidant capacity. Moreover, elevated RAR levels may also be linked to the development of neurodegenerative diseases, particularly Alzheimer’s disease and other types of cognitive impairment. Thus, RAR could serve as a potential and easily measurable biomarker for early identification of cognitive decline risk in older adults and for assessing their clinical prognosis. However, there are currently no studies directly confirming this relationship.

Although previous studies have independently linked RDW, ALB, and other inflammation-related biomarkers to cognitive impairment, these indicators typically reflect only a single physiological domain. In contrast, the RAR integrates both inflammatory and nutritional components, potentially offering greater sensitivity and specificity for identifying cognitive decline. However, to date, no study has systematically examined the association between RAR and cognitive impairment in a nationally representative population of older adults. Therefore, this study utilizes data from the National Health and Nutrition Examination Survey (NHANES) to investigate the relationship between RAR and cognitive impairment and to evaluate its potential as an early screening and risk stratification biomarker. The findings may provide novel insights for clinical practice and inform strategies for early intervention and prevention.

## Materials and methods

### Study design

This investigation utilized data from NHANES, a cross-sectional program that captures comprehensive health and nutritional insights from the U.S. civilian, non-institutionalized population. Since its inception in 1999, NHANES has operated on a biennial schedule to gather these metrics (GBD 2021 Causes of Death Collaborators, 2024). Approval for the survey was granted by the Research Ethics Review Board (Protocol #2011–14) at the National Center for Health Statistics, part of the CDC, and all adult subjects signed written informed consent forms. Data collection involved structured interviews, standardized clinical assessments, and laboratory analyses of biological specimens, including blood samples. The study was conducted based on a checklist and followed the STROBE (Strengthening the Reporting of Observational Studies in Epidemiology) guidelines to ensure rigor and transparency.

### Study population

**FIGURE 1:**
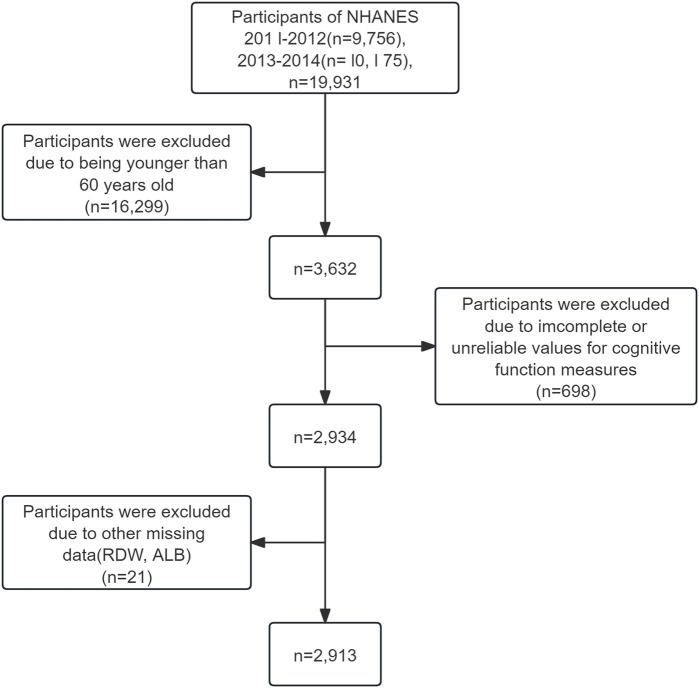
Selection of study population.

This study analyzed data from the 2011–2012 and 2013–2014 NHANES cycles, which were specifically selected because they were the only cycles that included all three cognitive evaluations: the Consortium to Establish a Registry for Alzheimer’s Disease (CERAD), the Animal Fluency Test (AFT), and the Digit Symbol Substitution Test (DSST). The research focused on individuals aged 60 and above who completed these cognitive assessments. Participants were excluded if any one of the five key variables—CERAD, AFT, DSST, RDW, or ALB—was missing. For other variables, those with more than 30% missing data were excluded using listwise deletion, while variables with less than 30% missingness were imputed using the random forest algorithm. In total, 2,913 individuals met the inclusion criteria and were incorporated into the final analysis (Figure 1).

### Measurement of RAR

The NHANES database employs specific methodologies for measuring serum albumin and RDW. Serum albumin levels are quantified using the bromocresol purple method, while RDW is assessed through the Coulter automated blood analyzer, which evaluates the variability in red blood cell volume distribution. RAR was calculated by dividing RDW (%) by ALB (g/dL), yielding a unitless ratio.

### Definition of cognitive impairment

In NHANES, cognitive function is evaluated using a series of standardized tools, including the CERAD Word Learning Subtest (CERAD W-L), the AFT, and the DSST. The CERAD W-L assesses memory by measuring both immediate and delayed recall of new verbal information (Morris et al., 1989). It consists of three consecutive learning trials and one delayed recall trial. In each learning trial, participants read aloud 10 unrelated words presented one at a time and immediately recall as many as possible. The sequence of words changes across trials, with a maximum score of 10 per trial. The AFT evaluates categorical verbal fluency, an aspect of executive function (Ma et al., 2020). Participants are tasked with naming as many animals as possible within 1 min, receiving a score for each correctly identified animal. This test has been validated for distinguishing individuals with normal cognition from those with mild cognitive impairment or advanced cognitive disorders, including Alzheimer’s disease (Clark et al., 2009; Georgakis et al., 2017; Canning et al., 2004). The DSST, part of the Wechsler Adult Intelligence Scale (WAIS-III), examines processing speed, sustained attention, and working memory. Participants are given 2 min to pair symbols with corresponding numbers in a grid containing 133 boxes. The total number of accurate matches determines the final score (Dori and Chelune, 2004).

Since no universally accepted gold standard cutoff exists for the CERAD, Animal Fluency, or DSST tests to define cognitive impairment, this study defined low cognitive performance as scores below the 25th percentile, calculated based on the distribution of test scores within our own study population, consistent with approaches adopted in previous literature (Chen et al., 2017). To account for the substantial influence of age on cognitive performance, scores were further stratified by age groups: 60 to <70 years, 70 to <80 years, and ≥80 years (Dong et al., 2020; Li et al., 2019). For the CERAD test, the cutoff scores indicating low cognitive ability were 22, 20, and 16 for the respective age groups. Similarly, the cutoff scores for the AFT were 14, 12, and 11, while for the DSST, they were 37, 32, and 28. Based on these thresholds, participants were classified into two categories for each test: the low cognitive ability group, comprising individuals scoring below the 25th percentile, and the normal cognitive ability group, consisting of those scoring above it.

### Covariates

A large number of covariates were included in this study. The selection of covariates was based on prior literature and theoretical considerations, aiming to control for potential confounders in the association between RAR and cognitive function. These included age, gender, height, weight, and race/ethnicity, categorized as non-Hispanic Black, non-Hispanic White, Mexican American, other Hispanic, and other races. Marital status was grouped into married/cohabiting, never married, and widowed/divorced/separated. Socioeconomic status was assessed using the poverty income ratio (PIR), divided into low income (<1.30), middle income (1.30–3.49), and high income (≥3.50). Education level was classified as less than high school, high school graduate, or college and above. Lifestyle factors included smoking status (never, former, and current smoker) and alcohol consumption, with drinking defined as consuming alcohol 12 or more times annually. Additionally, self-reported physician diagnosis medical history covered conditions such as hypertension, diabetes, depressed, sleep disorder, and other CVD.

### Statistical analysis

Data analysis was performed from 5 August 2024, to 21 October 2024. Descriptive statistics of participant characteristics were calculated using appropriate sampling weights to account for the NHANES survey design. Baseline characteristics were reported as frequencies (percentages) for categorical variables. For continuous variables, means and standard deviations (SD) were presented for data with approximately symmetric distributions, while medians and interquartile ranges (IQR) were used for skewed distributions. Comparisons between participants with and without cognitive impairment were conducted using independent sample t-tests for continuous variables and Rao-Scott χ^2^ tests for categorical variables. To investigate the association between RAR and cognitive impairment, three logistic regression models were developed: Model 1: Unadjusted. Model 2: Adjusted for age, race, education, marital status, weight, and height. Model 3 included all variables in Model 2, with additional adjustment for comorbidities (e.g., diabetes, hypertension, cardiovascular disease), PIR, smoking status, alcohol consumption.

Subgroup and interaction analyses were conducted to examine whether the association between RAR and cognitive impairment differed across various population groups, stratified by factors such as age, gender, race, education level, smoking status, alcohol consumption, hypertension, and CVD. These analyses were exploratory in nature. Although not pre-specified in the initial study design, they were grounded in prior literature and biological plausibility, and the findings may serve as a basis for hypothesis generation in future research. To assess potential nonlinear relationships between RAR and the risk of cognitive impairment, restricted cubic spline (RCS) regression was performed using 4 knots placed at the 5th, 35th, 65th, and 95th percentiles of RAR, following Harrell’s recommendations. The direct effects captured the relationship between RAR and cognitive impairment, while causal mediation analysis was utilized to estimate the proportion of the relationship mediated by specific factors. This methodology not only provides robust statistical support for mechanism exploration but also aids in identifying underlying biological pathways.

Causal mediation analysis was conducted using a generalized linear model with a probit link function, incorporating adjustments for confounding factors such as gender, age, race, and education level to account for covariate influences. Mediation effects were estimated via nonparametric bootstrap resampling with 1,000 iterations, and percentile-based confidence intervals were used to ensure reliability. The analysis quantified total effects, direct effects, and indirect effects mediated through specific pathways, calculating the proportion of the total effect attributable to mediators. To evaluate the moderating influence of RAR values, an interaction analysis (X*M) was performed, while residual correlations between the mediator and outcome models were assessed to exclude residual confounding.

All statistical analyses were carried out using Stata software (version 17.0, StataCorp) and the R programming environment (version 4.4.2, R Project for Statistical Computing). A two-sided (P < 0.05) was considered indicative of statistical significance.

## Results

### Characteristics of the study population

Table 1 presents the baseline characteristics of participants with and without cognitive impairment. Of the 2,913 participants included in the study, 1,291 (44.3%) were identified as having cognitive impairment, representing a significant proportion of individuals aged 60 years and older. The average age of participants was 69.69 ± 6.84 years, and 48.92% were male. Significant differences were observed between participants with and without cognitive impairment across several demographic variables, including age, gender, height, weight, race, education level, and marital status. Differences were also notable in biochemical markers such as globulin, albumin, lymphocyte ratio, hemoglobin, glycohemoglobin, and RDW. Additionally, medical history variables such as hypertension, diabetes, depression, CVD, stroke, cancer, as well as lifestyle factors like smoking and alcohol consumption, exhibited statistically significant disparities (P < 0.05). Detailed results are summarized in Table 1.

**TABLE 1:** Baseline characteristic of the study population.

Variables	Total (n = 2,913)	Non-cognitive impairment (n = 1,621)	Cognitive impairment (n = 1,292)	*P value*
Age, y	69.69 ± 6.84	69.44 ± 6.88	70.01 ± 6.78	0.025
Sex, n (%)				<0.001
Male	1,425 (48.92)	729 (44.97)	696 (53.87)	
Female	1,488 (51.08)	892 (55.03)	596 (46.13)	
Weight (kg)	79.55 ± 19.44	80.46 ± 19.69	78.39 ± 19.07	0.004
Hight (cm)	165.22 ± 9.99	165.70 ± 9.94	164.60 ± 10.03	0.004
Race, n (%)				<0.001
Mexican American	268 (9.20)	115 (7.09)	153 (11.84)	
Other Hispanic	296 (10.16)	102 (6.29)	194 (15.02)	
Non-Hispanic White	1,397 (47.96)	995 (61.38)	402 (31.11)	
Non-Hispanic Black	675 (23.17)	269 (16.59)	406 (31.42)	
Other Race - Including Multi-Racial	277 (9.51)	140 (8.64)	137 (10.60)	
Marital status, n (%)				<0.001
Married/Living with Partner	1,665 (57.16)	989 (61.01)	676 (52.32)	
Widowed/Divorced/Separated	1,075 (36.90)	549 (33.87)	526 (40.71)	
Never married	173 (5.94)	83 (5.12)	90 (6.97)	
Education levels, n (%)				<0.001
< High school	778 (26.71)	221 (13.63)	557 (43.11)	
High school	682 (23.41)	364 (22.46)	318 (24.61)	
College or above	1,453 (49.88)	1,036 (63.91)	417 (32.28)	
Income status				<0.001
High income	460 (17.23)	156 (10.39)	304 (26.05)	
Middle income	1,188 (44.51)	615 (40.95)	573 (49.10)	
Low income	1,021 (38.25)	731 (48.67)	290 (24.85)	
Albumin (g/L)	41.87 ± 3.11	42.09 ± 2.92	41.60 ± 3.31	<0.001
Globulin (g/L)	28.63 ± 4.91	27.78 ± 4.63	29.69 ± 5.05	<0.001
Total protein (g/L)	70.49 ± 4.97	69.86 ± 4.78	71.28 ± 5.11	<0.001
Lymphocyte percent (%)	28.91 ± 9.18	28.51 ± 8.84	29.42 ± 9.57	0.008
Hemoglobin (g/dL)	13.73 ± 1.46	13.87 ± 1.32	13.56 ± 1.60	<0.001
Glycohemoglobin (%)	6.08 ± 1.10	5.96 ± 0.90	6.23 ± 1.29	<0.001
RDW	13.63 ± 1.33	13.53 ± 1.24	13.76 ± 1.41	<0.001
RAR	3.28 ± 0.46	3.24 ± 0.42	3.34 ± 0.51	<0.001
Total Cholesterol (mg/dL)	190.93 ± 43.13	193.58 ± 43.11	187.60 ± 42.95	<0.001
CERAD	24.53 ± 6.77	27.84 ± 4.74	20.37 ± 6.65	<0.001
AST	16.42 ± 5.52	19.18 ± 4.68	12.96 ± 4.44	<0.001
DSST	45.95 ± 17.30	55.02 ± 13.12	33.96 ± 14.68	<0.001
Alcohol, n (%)				<0.001
No	532 (18.26)	228 (14.07)	304 (23.53)	
Yes	2,381 (81.74)	1,393 (85.93)	988 (76.47)	
High Blood Pressure, n (%)				0.003
No	1825 (62.65)	977 (60.27)	848 (65.63)	
Yes	1,088 (37.35)	644 (39.73)	444 (34.37)	
High Cholesterol Level, n (%)				0.345
No	1,632 (56.43)	925 (57.20)	707 (55.45)	
Yes	1,260 (43.57)	692 (42.80)	568 (44.55)	
Diabetes, n (%)				<0.001
No	2,127 (73.02)	1,255 (77.42)	872 (67.49)	
Yes	786 (26.98)	366 (22.58)	420 (32.51)	
Depressed, n (%)				<0.001
No	2,119 (73.88)	1,285 (80.01)	834 (66.09)	
Yes	749 (26.12)	321 (19.99)	428 (33.91)	
CVD, n (%)				0.002
No	2,398 (82.32)	1,366 (84.27)	1,032 (79.88)	
Yes	515 (17.68)	255 (15.73)	260 (20.12)	
Stroke, n (%)				<0.001
No	2,690 (92.50)	1,540 (95.18)	1,150 (89.15)	
Yes	218 (7.50)	78 (4.82)	140 (10.85)	
Cancer, n (%)				<0.001
No	2,340 (80.41)	1,244 (76.84)	1,096 (84.90)	
Yes	570 (19.59)	375 (23.16)	195 (15.10)	
Sleep Disorder, n (%)				0.131
No	2047 (70.30)	1,121 (69.15)	926 (71.73)	
Yes	865 (29.70)	500 (30.85)	365 (28.27)	
Smoke, n (%)				<0.001
Never	1,443 (49.54)	803 (49.54)	640 (49.54)	
Current	380 (13.04)	180 (11.10)	200 (15.48)	
Quitting smoking	1,090 (37.42)	638 (39.36)	452 (34.98)	

AFT, animal fluency test; CERAD, Consortium to Establish a Registry for Alzheimer’s Disease; CVD, cardiovascular disease; DSST, Digit symbol substitution test; RAR, red blood cell distribution width and albumin ratio; RDW, red cell distribution width.

Continuous variables were shown in mean (SD) and categorical variables were shown in percentages.

### Association between the RAR and cognitive impairment

Logistic regression analysis was performed to evaluate the association between RAR and cognitive function in elderly individuals, as detailed in Table 2. When RAR was treated as a continuous variable, the analysis revealed a positive association between RAR and the risk of cognitive impairment, indicating that higher RAR values corresponded to an increased likelihood of cognitive impairment (P < 0.05). When RAR was divided into quartiles and analyzed as a categorical variable, results across the unadjusted Model 1, partially adjusted Model 2, and fully adjusted Model 3 demonstrated no significant differences between the second, and third quartiles compared to the first quartile. However, Participants in the highest quartile had an OR of 1.33 (95% CI: 1.04∼1.72, P = 0.026) compared to the lowest quartile, suggesting a possible threshold effect. These findings highlight the potential role of elevated RAR levels in cognitive decline among the elderly population. RAR, as a composite marker of RDW and albumin, may reflect underlying systemic inflammation, oxidative stress, and poor nutritional status—factors that have been independently associated with cognitive decline. To further explore the dose–response relationship, we conducted an RCS regression analysis, which revealed a nonlinear association between RAR and cognitive impairment.

**TABLE 2:** Association of the RAR with cognitive impairment in the multivariate linear regression model.

Variables	Model 1		Model 2		Model 3	
	Or (95%CI)	*P*	Or (95%CI)	*P*	Or (95%CI)	*P*
RAR (continuous)	**1.62 (1.37 ∼ 1.91)**	**<0.001**	**1.24 (1.01 ∼ 1.53)**	**0.036**	**1.23 (1.01 ∼ 1.51)**	**0.048**
RAR (quartile)						
Quartile 1	1.00 (Reference)		1.00 (Reference)		1.00 (Reference)	
Quartile 2	1.00 (0.81–1.23)	0.999	0.97 (0.76–1.23)	0.785	0.95 (0.74–1.21)	0.663
Quartile 3	1.20 (0.97–1.47)	0.092	1.13 (0.89–1.44)	0.326	1.07 (0.84–1.37)	0.599
Quartile 4	**1.74 (1.41 ∼ 2.14)**	**<0.001**	**1.41 (1.10 ∼ 1.80)**	**0.007**	**1.33 (1.04 ∼ 1.72)**	**0.026**

CI, confidence interval; OR, Odds Ratio; RAR, red blood cell distribution width to albumin ratio.

Model 1: Crude.

Model 2: Adjust: Age, Education, Height, Marital status, Race, Weight.

Model 3: Adjust: Age, Cancer, Cardiovascular diseases, Depression, Diabetes, Education, Height, Hypertension, Marital status, Race, Sleep disorder, Stroke, Weight.

Note: Bold font denotes statistically significant differences.

### Dose-response analysis of RAR and cognitive impairment risk

**FIGURE 2:**
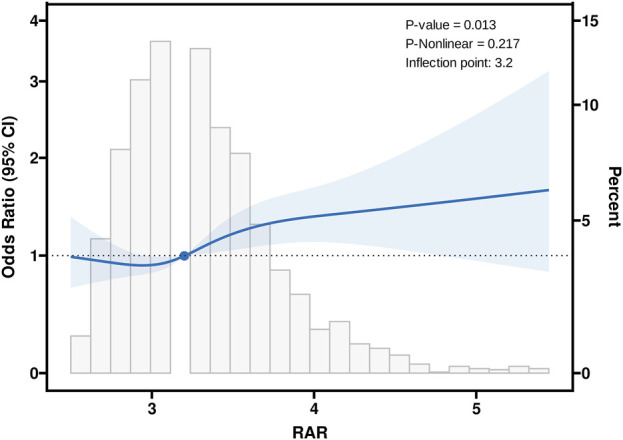
Association between RAR and Cognitive with the Restricted Cubic Spline function. RAR: Red Blood Cell Distribution Width to Albumin Ratio. Model with 4 knots located at 5th, 35th, 65th and 95th percentiles. Y-axis represents the OR to present Cognitive for any value of RAR compared to individuals with reference value (50th percentile) of RAR.

RCS analysis revealed a significant overall relationship between RAR and cognitive impairment (P = 0.013), as shown in Figure 2. The RCS curve identified an inflection point at RAR = 3.2, marking a critical threshold in the association between RAR levels and cognitive function. Based on this inflection point, participants were stratified into two groups: RAR<3.2 and RAR≥3.2. Segmented regression analysis was then performed for each group. The findings indicated that for individuals with RAR≥3.2, each one-standard-deviation increase in RAR was associated with a significantly higher risk of cognitive impairment (OR = 1.24, 95%CI:1.11–1.38, P < 0.001) (Table 3). In contrast, no significant association was observed between RAR and cognitive impairment for participants with RAR<3.2. These results suggest that elevated RAR levels (RAR≥3.2) may act as an independent risk factor for cognitive impairment. The identified threshold provides evidence for a potential nonlinear dose-response relationship, warranting further investigation into the mechanisms underlying the effect of RAR on cognitive health.

**TABLE 3:** Effect of standardized RAR level on cognitive: odds ratios from segmented logistic regression analysis.

Variables	N	OR per SD^a^	95% CI^a^	p-value
RAR (<3.2)	1,428	0.98	0.88, 1.09	0.75
RAR (≥3.2)	1,476	1.24	1.11, 1.38	<0.001

^a^ OR, odds ratio, SD: standard deviation, CI, confidence interval.

### Subgroup and stratified analysis of the association between RAR and cognitive impairment risk

**FIGURE 3:**
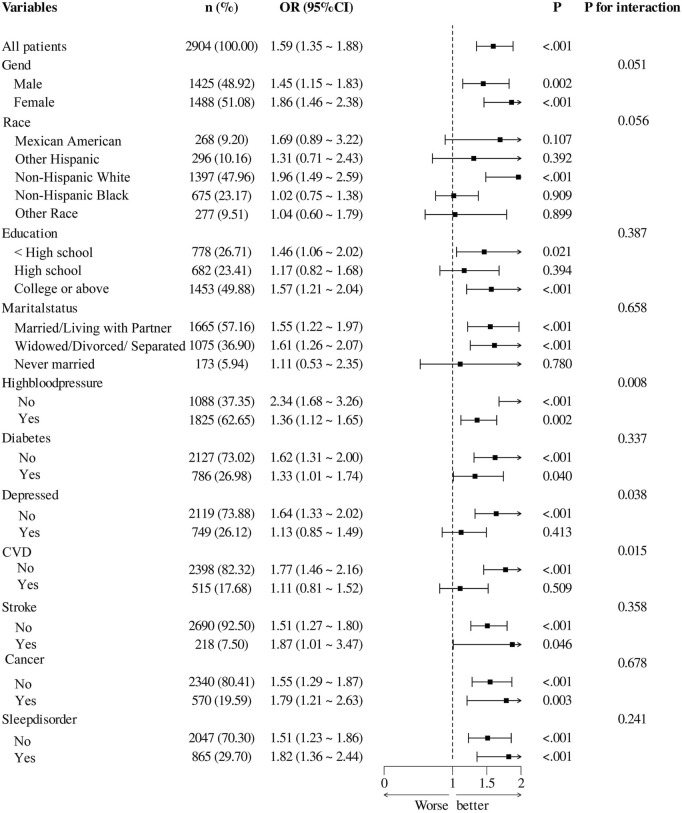
Associations Between Ratio of Red Blood Distribution Width to Albumin and cognitive impairment Among Subgroups CVD, cardiovascular disease.

Figure 3 presents the results of subgroup analyses investigating the association between RAR and cognitive impairment across diverse populations. Overall, elevated RAR levels were significantly associated with cognitive impairment (OR = 1.59, 95% CI: 1.35–1.88, P < 0.001). A significant interaction was observed in the hypertension subgroup (P for interaction = 0.008), with a stronger association in individuals without hypertension (OR = 2.34, 95% CI: 1.68–3.26, P < 0.001) compared to those with hypertension (OR = 1.36, 95% CI: 1.12–1.65, P = 0.002). Similarly, significant interaction effects were identified in the depression (P = 0.038) and CVD subgroups (P = 0.015), with more pronounced associations observed among participants without these comorbidities. No significant interaction effects were found across subgroups stratified by sex, race, educational level, marital status, diabetes, stroke, cancer, or sleep disorders. For subgroups with significant interaction effects, further stratified analyses were performed to assess trends in odds ratios (ORs) and confidence intervals across population groups (Supplementary Tables S1–S6). In addition, multivariable models combined with RCS analyses revealed significant nonlinear relationships between RAR and cognitive impairment in individuals without hypertension, CVD, or depression (Supplementary Figures S1–S6). These findings suggest that the association between RAR and cognitive impairment may be modified by specific health conditions.

### Causal mediation analysis in the relationship between RAR and the risk of cognitive impairment

**FIGURE 4:**
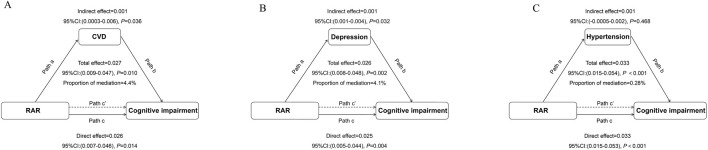
Path Diagram of the Mediation Analysis Model for the Relationship Between RAR and Cognitive Impairment **(A)** cardiovascular disease (CVD); **(B)**. Depression; **(C)**. Hypertension. In the mediation analysis, RAR is defined as the exposure factor, cognitive impairment as the outcome variable, and CVD as the mediator. Path a represents the regression coefficient for the association between RAR and the mediator. Path b represents the regression coefficient for the association between the mediator and cognitive impairment. Path c represents the simple total effect of RAR on cognitive impairment, unadjusted for the mediator’s influence. Path c’ represents the direct effect of RAR on cognitive impairment after controlling for the mediator’s influence. The adjusted confounding factors included age, race, education level, marital status, weight, height, diabetes, stroke, cancer, and sleep disorders.

Causal mediation analysis evaluated the impact of RAR on cognitive impairment, using CVD, depression, and hypertension as mediators. When CVD was considered as a mediator, the estimated mediation effect of RAR on cognitive impairment through CVD was 0.001 (95% CI: 0.0003, 0.004, P = 0.036), suggesting a modest but statistically significant indirect effect through the CVD pathway. The proportion of the total effect mediated by CVD was estimated at 4.49% (Figure 4A). When depression was used as a mediator, the estimated mediation effect of RAR on cognitive impairment through depression was 0.001 (95% CI: 0.001, 0.004, P = 0.032), with the proportion of the total effect mediated by depression estimated at 4.1% (Figure 4B). For hypertension as a mediator, the mediation effect of RAR on cognitive impairment through hypertension was 0.001 (95% CI: 0.0005, 0.02, P = 0.468), indicating that hypertension did not serve as a mediator in the relationship between RAR and cognitive function (Figure 4C). The interaction tests (X*M interaction) between RAR and cardiovascular disease, depression, and hypertension showed that the correlation between the residuals of the mediator model and the outcome model was less than 0.05, suggesting no unmeasured confounding in the exposure–mediator and mediator–outcome relationships.

## Discussion

This large-scale cross-sectional study analyzed data from NHANES, concentrating on Americans aged 60 years and older. Using three cognitive function assessments—CERAD, AFT, and DSST—the study identified a statistically significant association between RAR and cognitive impairment. The results demonstrated that higher RAR levels, particularly RAR ≥3.2, are robust association to an increased prevalence of cognitive impairment in older adults. The RCS analysis identified an inflection point at RAR = 3.2, suggesting a potential threshold above which the risk of cognitive impairment may increase more sharply. Although this specific value has not been previously established in earlier literature, it is biologically plausible. Elevated RDW, a component of RAR, reflects increased systemic inflammation and oxidative stress, while decreased albumin indicates malnutrition and reduced anti-inflammatory capacity—both strongly linked to cognitive dysfunction. The fact that individuals with RAR ≥3.2 in our analysis exhibited significantly higher cognitive impairment prevalence further supports the potential clinical relevance of this threshold. Therefore, RAR = 3.2 may serve as a meaningful indicator for clinical risk stratification. Further studies, particularly prospective cohorts, are needed to validate this cutoff and determine its applicability in diverse populations.

This finding supports the hypothesis that inflammatory states and metabolic abnormalities are involved in the development of cognitive impairment. As an integrated indicator combining RDW and ALB, RAR reflects both systemic inflammatory burden and changes in nutritional status. Elevated RDW is often associated with inflammatory responses and oxidative stress (Hong et al., 2019; Jiang et al., 2021; Li et al., 2024; Song and Lee, 2020), mechanisms that may directly impair cognitive function through microcirculatory disturbances, cerebral small vessel disease, and neuroinflammation (Hassan et al., 2015; Yu et al., 2024). Meanwhile, reduced albumin levels indicate a weakened anti-inflammatory and antioxidant capacity, further exacerbating cerebral inflammation and oxidative stress. Elevated RAR may play a dual role in the development of cognitive impairment. On one hand, the persistent activation of inflammatory factors can lead to vascular endothelial dysfunction in the brain, impairing blood flow and oxygen delivery to brain tissue, thereby accelerating neuronal degeneration (Daiber et al., 2019; Gu et al., 2021; Wang et al., 2019). In contrast, increased RAR may drive the pathological progression of neurodegenerative diseases such as Alzheimer’s disease by altering metabolic homeostasis and promoting the formation of inflammasomes (Liu et al., 2014; McManus and Latz, 2024; Zhuang et al., 2015). Particularly in the elderly population, the accumulation of systemic inflammation and metabolic imbalance with aging may significantly enhance the predictive value of RAR for cognitive impairment.

Subgroup analysis demonstrated significant interactions between depression, hypertension, and CVD in the relationship between RAR and cognitive impairment (P for interaction<0.05). These findings suggest that these factors modulate the effect of RAR on cognitive function across different subgroups. Further stratified analysis revealed a significant linear relationship between RAR and cognitive function in non-hypertensive, non-CVD, and non-depressed participants. This finding may indicate that, in these subgroups, RAR serves as a more direct and reliable biomarker for predicting cognitive impairment. One possible explanation is that, in individuals without hypertension, CVD, or depression, the effects of inflammation and nutritional imbalance—as reflected by RAR—may be more pronounced and less confounded by other dominant pathological processes. In contrast, among those with such comorbidities, factors such as chronic vascular remodeling, neuroinflammatory cascades, or long-standing endothelial dysfunction may exert stronger influences on cognitive function, thereby attenuating or obscuring the impact of RAR (Cai et al., 2021; Hall et al., 2021; Zhang et al., 2023). Chronic vascular remodeling, characterized by arterial stiffness and atherosclerotic plaque formation, can lead to reduced cerebral perfusion and disruption of neurovascular coupling, ultimately resulting in chronic cerebral hypoxia and white matter lesions. Neuroinflammatory cascades, often triggered by systemic diseases, involve activation of microglia and astrocytes, the release of pro-inflammatory cytokines such as IL-1β and TNF-α, and increased oxidative stress—all of which collectively impair synaptic function and neuronal integrity (Bairamian et al., 2022; Tastan and Heneka, 2024; Xu et al., 2023). Endothelial dysfunction, a hallmark of both hypertension and CVD, may result in reduced nitric oxide bioavailability, increased leukocyte adhesion, and enhanced blood-brain barrier permeability, thereby exacerbating neurodegenerative processes (Andjelkovic et al., 2023; Sweeney et al., 2018). These chronic pathological mechanisms may dominate the clinical presentation, potentially masking the modest effects of RAR in individuals with significant comorbidities. Furthermore, commonly prescribed medications for hypertension, CVD, and depression—such as ACE inhibitors, statins, and selective serotonin reuptake inhibitors (SSRIs)—may alter systemic inflammatory and metabolic states, further attenuating the direct impact of RAR (Guo et al., 2023; Pan et al., 2023). These notable differences across subgroups highlight the heterogeneity in the relationship between RAR and cognitive function and underscore the importance of considering interaction effects within specific disease contexts.

The stronger linear relationship between RAR and cognitive impairment observed in individuals without hypertension, CVD, or depression may reflect the absence of confounding from overlapping pathologies or medication effects. In these populations, RAR likely captures systemic inflammation and nutritional status more directly. Without the vascular remodeling, neurochemical changes, or pharmacologic interventions common in comorbid individuals, the pathophysiological link between RAR and cognitive function may be more clearly expressed. This highlights the potential utility of RAR as a more sensitive biomarker in relatively healthy older adults, and underscores the importance of disease context when interpreting biomarker effects. This observation suggests that RAR may serve as a potential biomarker for predicting cognitive impairment in older adults, particularly among those without major comorbidities. Second, the linear association provides a rationale for defining RAR thresholds and guiding risk stratification, which could facilitate the early identification of high-risk populations. Furthermore, this finding underscores the potential utility of RAR as a screening tool in public health settings. As an inexpensive and widely available parameter, the observed linearity supports its use as a preliminary risk assessment tool, even in resource-limited healthcare environments. Such an approach may inform triage strategies and enable early detection of individuals at increased risk of cognitive decline. While not intended to replace diagnostic methods, the use of RAR as a blood-based indicator could enhance early-stage screening efforts and promote timely intervention, particularly in underserved populations.

Causal mediation analysis further revealed that CVD and depression play significant roles in the relationship between RAR and cognitive impairment, while hypertension showed no significant mediating effect. Among these mediators, CVD accounted for a larger proportion of the mediation effect compared to depression, suggesting that it may be the primary modulating factor in the influence of RAR on cognitive function. CVD may indirectly affect cognitive function through multiple pathways: on one hand, systemic hypoxia, endothelial dysfunction, and chronic inflammation induced by CVD can directly impair cerebral blood flow and neuronal health (Chen, 2023; Iadecola et al., 2019; Stephan et al., 2017). In contrast, these pathological changes may alter RDW and ALB in the blood, thereby increasing the RAR value (Fu et al., 2023; He et al., 2024). In contrast, while depression also acts as an important mediator, its effects are likely concentrated on neurotransmitter imbalances, elevated levels of inflammatory factors, and psychological and behavioral changes (Berk et al., 2013; Beurel et al., 2020; Zhao et al., 2019) These mechanisms collectively explain the multidimensional pathways through which RAR indirectly impacts cognitive function via CVD and depression. Although the mediation effects of CVD (4.49%) and depression (4.1%) were relatively modest, they were statistically significant and may still carry clinical relevance. Even small-to-moderate mediation proportions can represent biologically plausible pathways in the context of cognitive impairment, a multifactorial and complex condition. In large populations, such modest indirect effects may still translate into meaningful public health burdens. These findings also point to potential cardiovascular and neuropsychiatric susceptibility subgroups, which may serve as targets for early intervention. Nonetheless, we acknowledge that the modest magnitude of these mediation effects limits their utility as standalone predictive factors. Therefore, RAR is best interpreted as part of a multifactorial risk assessment framework.

This study, by combining subgroup analysis and causal mediation analysis, provides an in-depth understanding of the complex regulatory mechanisms linking RAR and cognitive impairment. The significant interaction effects and mediating roles underscore the importance of considering comorbid conditions when studying the impact of RAR on cognitive function. The identification of CVD and depression as key mediators offers new insights into the role of RAR in brain health and points to potential directions for clinical intervention strategies. Future research should further explore the causal pathways between RAR and cognitive impairment under specific disease contexts, incorporating additional biomarkers such as inflammatory factors and brain imaging indices to develop more comprehensive predictive models. Moreover, clinical trials could validate whether interventions targeting CVD and depression can effectively improve RAR levels and slow the progression of cognitive impairment, providing stronger evidence for personalized treatment. These studies will hold profound implications for improving the prevention and management of cognitive impairment in the elderly.

## Limitations

Although this study only analyzed data from the 2011–2012 and 2013–2014 NHANES cycles, it is important to note that NHANES employs a complex, multistage probability sampling design intended to yield a nationally representative sample of the non-institutionalized U.S. population. Thus, the findings are statistically generalizable to older adults in the United States, albeit with certain limitations. First, the cross-sectional design precludes causal inference between RAR and cognitive impairment. Second, although RAR is a simple and easily measurable biomarker with clinical potential, it may not be entirely specific to cognitive decline. RAR levels can be influenced by multiple confounding factors, including hepatic dysfunction, systemic inflammation, malnutrition, and underlying chronic diseases. Additionally, unmeasured confounders and the reliance on self-reported data for certain variables may affect the robustness of our findings. Therefore, future longitudinal studies are warranted to account for these potential sources of bias and to further validate the clinical utility of RAR.

## Conclusion

RAR is significantly associated with cognitive impairment in elderly patients, with a stronger correlation observed when RAR ≥3.2. Both CVD and depression not only influence but also partially mediate this relationship. These findings suggest that RAR could serve as a potential biomarker and screening tool for cognitive impairment, particularly among elderly individuals without major comorbidities, where the nonlinear association appears more evident. Nevertheless, longitudinal studies are warranted to validate the predictive value of RAR and explore its clinical utility in early identification and prevention strategies.

## Data Availability

Publicly available datasets were analyzed in this study. This data can be found here: All data can be found at https://wwwn.cdc.gov/nchs/nhanes/Default.aspx.
