# Supplementary material for: Cardiovascular disease and depression as mediators between red blood cell distribution width to albumin ratio and cognitive impairment in older adults
Source: Front Physiol. 2025 Jun 11;16:1587635. doi: 10.3389/fphys.2025.1587635 (PMC12187606; doi:10.3389/fphys.2025.1587635)
Supplement: Supplementary file 1 [file DataSheet1.docx]

**Supplement material legend:**

Supplement table 1. Multi-model analysis of RAR and cognitive impairment in elderly patients with cardiovascular disease.

Supplement table 2. Multi-model analysis of RAR and cognitive impairment in elderly patients with non-cardiovascular diseases.

Supplement table 3. Multi-model analysis of RAR and cognitive impairment in elderly patients with hypertension.

Supplement table 4. Multi-model analysis of RAR and cognitive impairment in elderly patients with non-hypertension.

Supplement table 5. Multi-model analysis of RAR and cognitive impairment in elderly patients with depressed.

Supplement table 6. Multi-model analysis of RAR and cognitive impairment in elderly patients with non-depressed.

Supplement Figure 1. Restricted Cubic Spline curve predicting the relationship between RAR and cognitive impairment in elderly patients with cardiovascular diseases.

Supplement Figure 2. Restricted Cubic Spline curve predicting the relationship between RAR and cognitive impairment in elderly patients with hypertensive.

Supplement Figure 3. Restricted Cubic Spline curve predicting the relationship between RAR and cognitive impairment in elderly patients with depression.

Supplement table 1. Multi-model analysis of RAR and cognitive impairment in elderly patients with cardiovascular disease.

| Variables | Model 1 | | Model 2 | | Model 3 | |
| --- | --- | --- | --- | --- | --- | --- |
|  | OR (95%CI) | *P* | OR (95%CI) | *P* | OR (95%CI) | *P* |
| RAR (continuous) | 1.11 (0.81 ~ 1.52) | 0.499 | 0.64 (0.35 ~ 1.16) | 0.142 | 0.65 (0.36 ~ 1.19) | 0.162 |
| RAR (quartile) |  |  |  |  |  |  |
| Quartile 1 | 1.00 (Reference) |  | 1.00 (Reference) |  | 1.00 (Reference) |  |
| Quartile 2 | 0.77 (0.44 ~ 1.35) | 0.362 | 0.79 (0.41 ~ 1.55) | 0.500 | 0.77 (0.39 ~ 1.54) | 0.464 |
| Quartile 3 | 0.87 (0.52 ~ 1.47) | 0.604 | 0.91 (0.46 ~ 1.80) | 0.796 | 0.87 (0.43 ~ 1.75) | 0.692 |
| Quartile 4 | 1.27 (0.77 ~ 2.09) | 0.341 | 1.45 (0.59 ~ 3.56) | 0.415 | 1.47 (0.59 ~ 3.70) | 0.410 |

RAR: Red Blood Cell Distribution Width to Albumin Ratio

OR: Odds Ratio, CI: Confidence Interval

Model1: Crude

Model2: Adjust: age, Race, Education, Marital status, weight, height

Model3: Adjust: age, Race, Education, Marital status, weight, Hight, Diabetes, stroke, Cancer, Sleep Disorder, Depressed, Hypertension.

Supplement table 2. Multi-model analysis of RAR and cognitive impairment in elderly patients with non-cardiovascular diseases.

| Variables | Model 1 | | Model 2 | | Model 3 | |
| --- | --- | --- | --- | --- | --- | --- |
|  | OR (95%CI) | P | OR (95%CI) | P | OR (95%CI) | P |
| RAR (continuous) | **1.79 (1.47 ~ 2.18)** | **<.001** | **1.55 (1.22 ~ 1.97)** | **<.001** | **1.42 (1.11 ~ 1.81)** | **0.006** |
| RAR (quartile) |  |  |  |  |  |  |
| Quartile 1 | 1.00 (Reference) |  | 1.00 (Reference) |  | 1.00 (Reference) |  |
| Quartile 2 | 1.04 (0.83 ~ 1.31) | 0.736 | 0.99 (0.76 ~ 1.28) | 0.933 | 0.94 (0.72 ~ 1.22) | 0.643 |
| Quartile 3 | 1.25 (0.99 ~ 1.56) | 0.058 | 1.20 (0.92 ~ 1.56) | 0.179 | 1.15 (0.87 ~ 1.51) | 0.325 |
| Quartile 4 | **1.78 (1.41 ~ 2.25)** | **<.001** | **1.52 (1.15 ~ 2.01)** | **0.003** | **1.37 (1.03 ~ 1.82)** | **0.031** |

RAR: Red Blood Cell Distribution Width to Albumin Ratio

OR: Odds Ratio, CI: Confidence Interval

Model1: Crude

Model2: Adjust: age, Race, Education, Marital status, weight, height

Model3: Adjust: age, Race, Education, Marital status, weight, Hight, Diabetes, stroke, Cancer, Sleep Disorder, Depressed, Hypertension.

Supplement table 3. Multi-model analysis of RAR and cognitive impairment in elderly patients with hypertension.

| Variables | Model 1 | | Model 2 | | Model 3 | |
| --- | --- | --- | --- | --- | --- | --- |
|  | OR (95%CI) | P | OR (95%CI) | P | OR (95%CI) | P |
| RAR (continuous) | **1.37 (1.14 ~ 1.66)** | **0.001** | 1.15 (0.91 ~ 1.44) | 0.235 | 1.07 (0.85 ~ 1.35) | 0.559 |
| RAR (quartile) |  |  |  |  |  |  |
| Quartile 1 | 1.00 (Reference) |  | 1.00 (Reference) |  | 1.00 (Reference) |  |
| Quartile 2 | 0.87 (0.66 ~ 1.14) | 0.299 | 0.83 (0.61 ~ 1.13) | 0.227 | 0.76 (0.56 ~ 1.05) | 0.095 |
| Quartile 3 | 0.98 (0.75 ~ 1.29) | 0.901 | 0.96 (0.70 ~ 1.32) | 0.816 | 0.88 (0.64 ~ 1.22) | 0.451 |
| Quartile 4 | **1.38 (1.07 ~ 1.79)** | **0.015** | 1.15 (0.84 ~ 1.56) | 0.387 | 1.04 (0.76 ~ 1.43) | 0.801 |

RAR: Red Blood Cell Distribution Width to Albumin Ratio

OR: Odds Ratio, CI: Confidence Interval

Model1: Crude

Model2: Adjust: age, Race, Education, Marital status, weight, height

Model3: Adjust: age, Race, Education, Marital status, weight, Hight, Diabetes, stroke, Cancer, Sleep Disorder, Depressed, cardiovascular diseases.

Supplement table 4. Multi-model analysis of RAR and cognitive impairment in elderly patients with non-hypertension.

| Variables | Model 1 | | Model 2 | | Model 3 | |
| --- | --- | --- | --- | --- | --- | --- |
|  | OR (95%CI) | P | OR (95%CI) | P | OR (95%CI) | P |
| RAR (continuous) | **2.32 (1.68 ~ 3.21)** | **<.001** | **2.00 (1.35 ~ 2.95)** | **<.001** | **1.87 (1.25 ~ 2.79)** | **0.002** |
| RAR (quartile) |  |  |  |  |  |  |
| Quartile 1 | 1.00 (Reference) |  | 1.00 (Reference) |  | 1.00 (Reference) |  |
| Quartile 2 | 1.21 (0.87 ~ 1.70) | 0.262 | 1.15 (0.79 ~ 1.70) | 0.466 | 1.16 (0.78 ~ 1.72) | 0.467 |
| Quartile 3 | **1.56 (1.12 ~ 2.18)** | **0.009** | 1.42 (0.96 ~ 2.09) | 0.077 | 1.36 (0.91 ~ 2.03) | 0.128 |
| Quartile 4 | **2.43 (1.69 ~ 3.49)** | **<.001** | **2.09 (1.36 ~ 3.21)** | **<.001** | **1.92 (1.23 ~ 2.99)** | **0.004** |

RAR: Red Blood Cell Distribution Width to Albumin Ratio

OR: Odds Ratio, CI: Confidence Interval

Model1: Crude

Model2: Adjust: age, Race, Education, Marital status, weight, height

Model3: Adjust: age, Race, Education, Marital status, weight, Hight, Diabetes, stroke, Cancer, Sleep Disorder, Depressed, cardiovascular diseases.

Supplement table 5. Multi-model analysis of RAR and cognitive impairment in elderly patients with depressed.

| Variables | Model 1 | | Model 2 | | Model 3 | |
| --- | --- | --- | --- | --- | --- | --- |
|  | OR (95%CI) | P | OR (95%CI) | P | OR (95%CI) | P |
| RAR (continuous) | 1.14 (0.86 ~ 1.51) | 0.357 | 1.06 (0.76 ~ 1.49) | 0.732 | 1.07 (0.76 ~ 1.51) | 0.707 |
| RAR (quartile) |  |  |  |  |  |  |
| Quartile 1 | 1.00 (Reference) |  | 1.00 (Reference) |  | 1.00 (Reference) |  |
| Quartile 2 | 0.79 (0.51 ~ 1.23) | 0.292 | 0.87 (0.53 ~ 1.45) | 0.603 | 0.85 (0.51 ~ 1.41) | 0.523 |
| Quartile 3 | 1.07 (0.68 ~ 1.67) | 0.771 | 1.08 (0.64 ~ 1.83) | 0.779 | 1.03 (0.60 ~ 1.76) | 0.927 |
| Quartile 4 | 1.16 (0.77 ~ 1.77) | 0.476 | 1.20 (0.72 ~ 1.98) | 0.484 | 1.19 (0.71 ~ 2.00) | 0.508 |

RAR: Red Blood Cell Distribution Width to Albumin Ratio

OR: Odds Ratio, CI: Confidence Interval

Model1: Crude

Model2: Adjust: age, Race, Education, Marital status, weight, height

Model3: Adjust: age, Race, Education, Marital status, weight, Hight, Diabetes, stroke, Cancer, Sleep Disorder, cardiovascular diseases, Hypertension.

Supplement table 6. Multi-model analysis of RAR and cognitive impairment in elderly patients with non-depressed.

| Variables | Model 1 | | Model 2 | | Model 3 | |
| --- | --- | --- | --- | --- | --- | --- |
|  | OR (95%CI) | P | OR (95%CI) | P | OR (95%CI) | P |
| RAR (continuous) | **1.68 (1.36 ~ 2.06)** | **<.001** | **1.37 (1.07 ~ 1.75)** | **0.012** | **1.29 (1.01 ~ 1.66)** | **0.045** |
| RAR (quartile) |  |  |  |  |  |  |
| Quartile 1 | 1.00 (Reference) |  | 1.00 (Reference) |  | 1.00 (Reference) |  |
| Quartile 2 | 1.02 (0.79 ~ 1.30) | 0.895 | 0.96 (0.73 ~ 1.27) | 0.774 | 0.95 (0.72 ~ 1.25) | 0.712 |
| Quartile 3 | 1.18 (0.93 ~ 1.50) | 0.184 | 1.14 (0.87 ~ 1.51) | 0.338 | 1.11 (0.84 ~ 1.47) | 0.455 |
| Quartile 4 | **1.68 (1.31 ~ 2.16)** | **<.001** | **1.35 (1.01 ~ 1.82)** | **0.043** | **1.36 (1.01 ~ 1.82)** | **0.043** |

RAR: Red Blood Cell Distribution Width to Albumin Ratio

OR: Odds Ratio, CI: Confidence Interval

Model1: Crude

Model2: Adjust: age, Race, Education, Marital status, weight, height

Model3: Adjust: age, Race, Education, Marital status, weight, Hight, Diabetes, stroke, Cancer, Sleep Disorder, cardiovascular diseases, Hypertension.

Supplement Figure 1. Restricted Cubic Spline curve predicting the relationship between RAR and cognitive impairment in elderly cardiovascular patients.
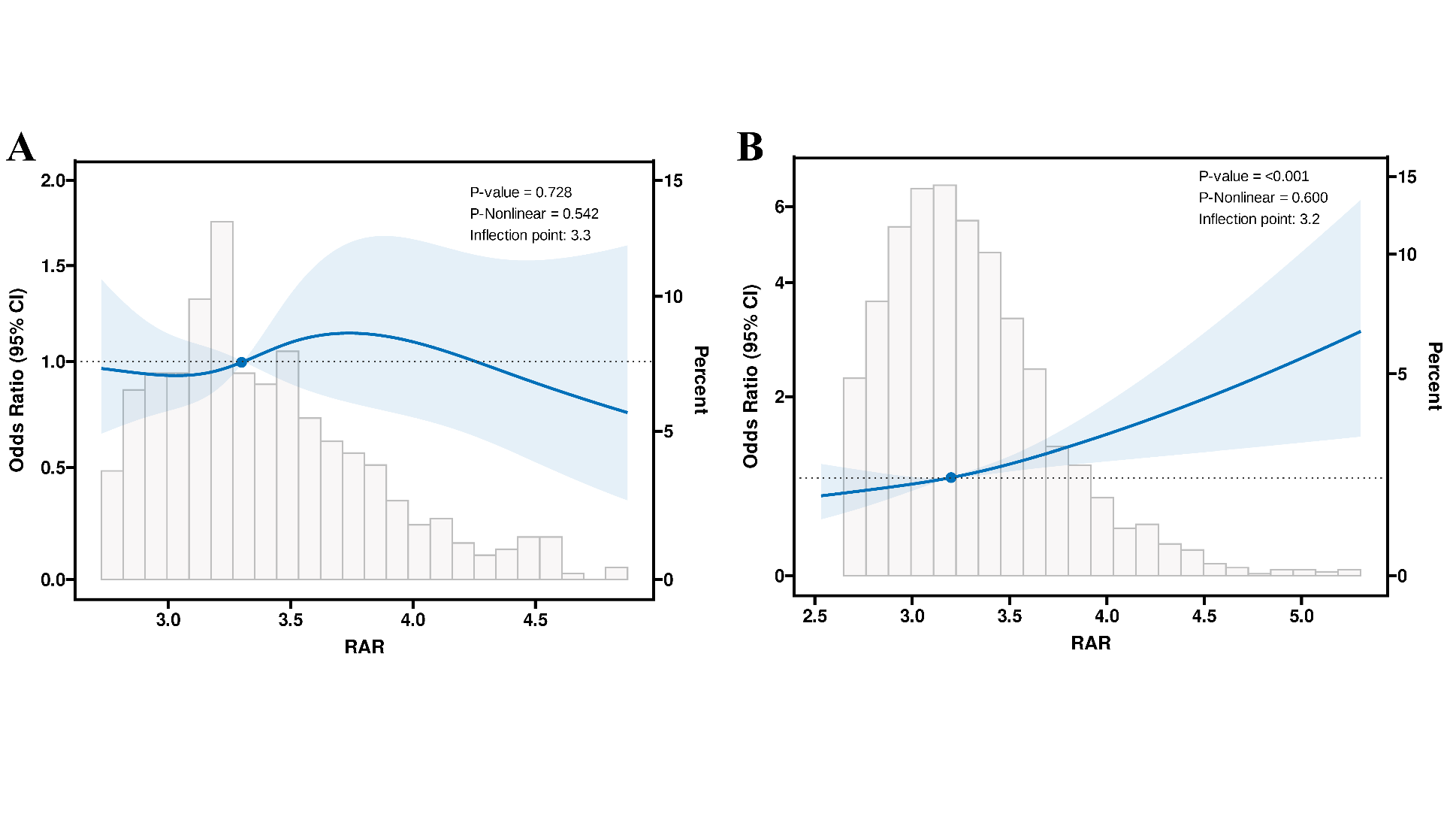


A. Cardiovascular patients, B. Non-cardiovascular patients

Supplement Figure 2. Restricted Cubic Spline curve predicting the relationship between RAR and cognitive impairment in elderly hypertensive patients.
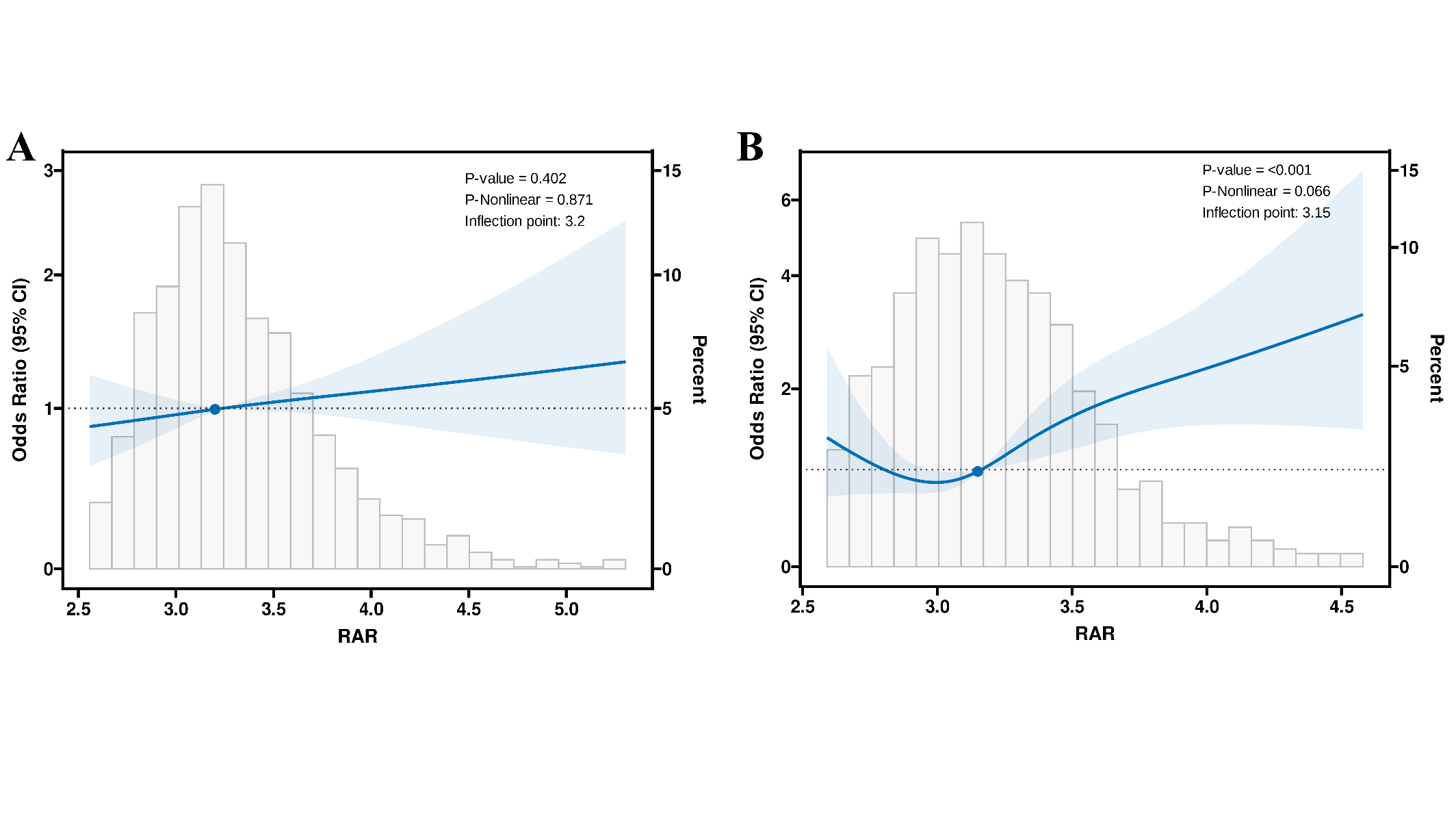


A. Hypertensive patients, B. Non-hypertensive patients

Supplement Figure 3. Restricted Cubic Spline curve predicting the relationship between RAR and cognitive impairment in elderly patients with depression.


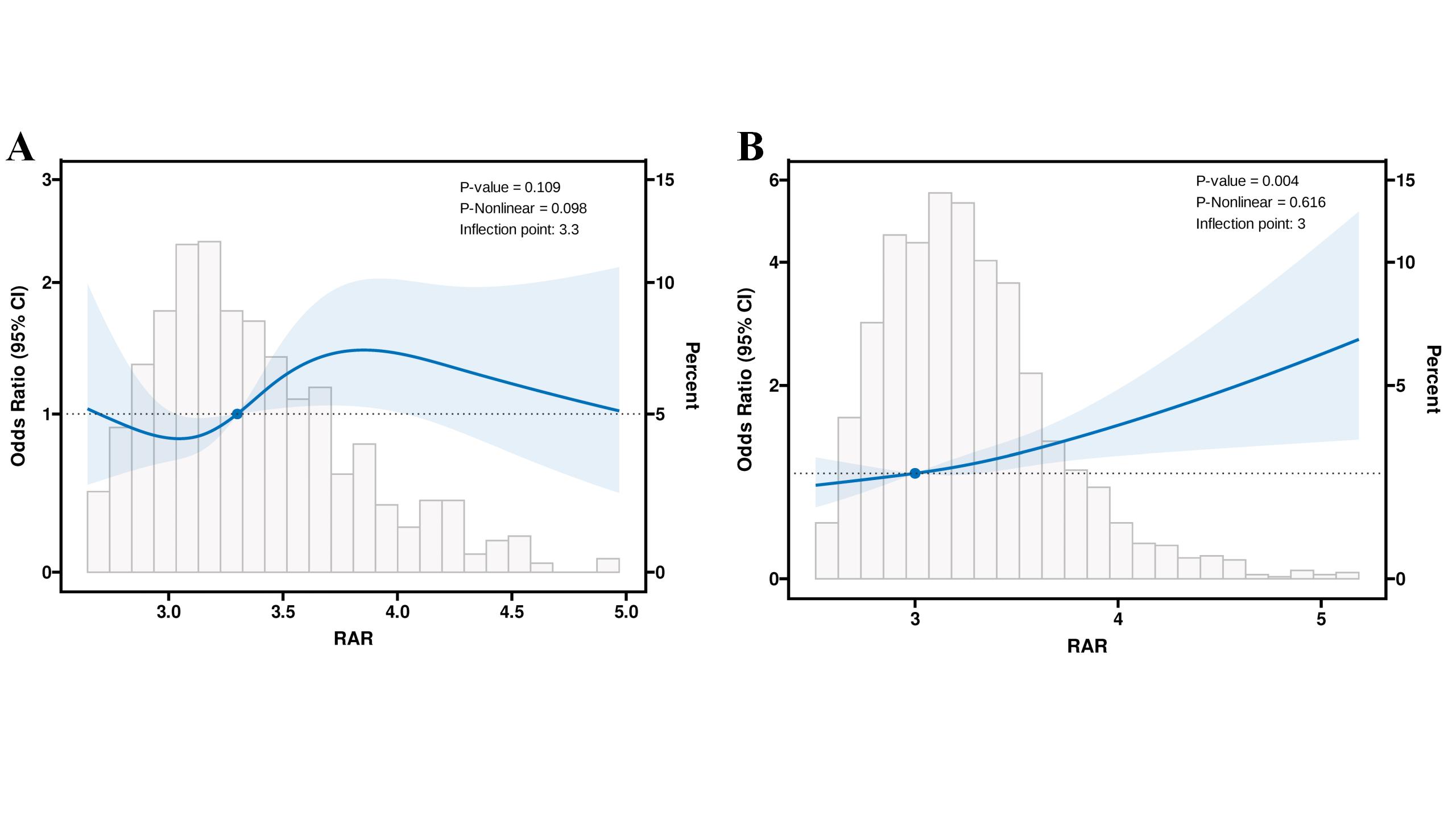


A. Depressed patients, B. Non-depressed patients
